# Supplementary material for: Herpes Simplex Virus Infection Alters the Immunological Properties of Adipose-Tissue-Derived Mesenchymal-Stem Cells
Source: Int J Mol Sci. 2023 Jul 26;24(15):11989. doi: 10.3390/ijms241511989 (PMC10418794; doi:10.3390/ijms241511989)
Supplement: Supplementary file 1 [file ijms-24-11989-s001.zip › ijms-2378729-supplementary.pdf]

Herpes Simplex Virus Infection Alters the Immunological Properties of Adipose-Tissue-Derived Mesenchymal Stem-like Cells

Anikó Kun-Varga[1,2#], Barbara Gubán[1#], Vanda Miklós[3], Shahram Parvaneh[4], Melinda Guba[1,5], Szűcs Diána[1,5], Tamás Monostori[1,5], János Varga[6], Ákos Varga[6], Zsolt Rázga[7†], Zsuzsanna Bata-Csörgő[4], Lajos Kemény[1,4,5], Klára Megyeri[8], Zoltán Veréb[1,3,5*]

Supplementary Information

Materials and methods

Differentiation

To do the trilineage differentiation. after the trypsinization of the cells. they were counted using EVE automatic cell counter. NanoEntek (NanoEntek. Seoul. Korea) and 50.000 cells were seeded in 24-well plates. The isolated cells were applied to commercially available Gibco's StemPro® Adipogenesis. Osteogenesis. and Chondrogenesis Differentiation Kits according to the manufacturer's guidelines (Gibco). After 24 hours. the medium was replaced with a differentiation medium. After 21 days. cells were fixed with 4% methanol-free formaldehyde (Molar Chemicals. Hungary) for 20 min at RT. In the differentiated adipocytes. Nile red staining was used to detect the lipid-laden particles. Alizarin red staining was applied to quantify the mineral deposits during osteogenesis. And Toluidine blue staining was used to label the chondrogenic mass formed by AD-MSC.

RNA-seq analysis

enrichplot: Yu G (2022). *enrichplot: Visualization of Functional Enrichment Result*. R package version 1.16.1. https://yulab-smu.top/biomedical-knowledge-mining-book/.

GOSemSim:
- Guangchuang Yu. Gene Ontology Semantic Similarity Analysis Using GOSemSim. In: Kidder B. (eds) Stem Cell Transcriptional Networks. Methods in Molecular Biology. 2020. 2117:207-215. Humana. New York. NY. doi:10.1007/978-1-0716-0301-7_11
- Guangchuang Yu. Fei Li. Yide Qin. Xiaochen Bo. Yibo Wu. Shengqi Wang. GOSemSim: an R package for measuring semantic similarity among GO terms and gene products Bioinformatics 2010. 26(7):976-978. doi:10.1093/bioinformatics/btq064

rrvgo:
Sayols S (2020). *rrvgo: a Bioconductor package to reduce and visualize Gene Ontology terms*. https://ssayols.github.io/rrvgo.

EnhancedVolcano:
Blighe K. Rana S. Lewis M (2022). *EnhancedVolcano: Publication-ready volcano plots with enhanced coloring and labeling*. R package version 1.14.0

Donor	Age	Sex (M/F)
1	40	M
2	35	M
3	34	M
4	47	M
5	51	F
6	53	F
7	42	F
8	25	M
9	65	F
10	36	M
11	46	F
12	53	M
13	41	F
14	39	F

Table S1. Details of donors used in this study.

Surface marker	Mean (% of positive cells)	SEM (+/-)
CD6	2.58	0.88
CD29	***98.74***	***0.25***
CD31	0.05	0.03
CD34	***0.00***	***0.00***
CD40	3.14	2.35
CD44	***90.39***	***2.15***
CD47	95.79	0.64
CD49A	93.61	1.51
CD49D	29.04	13.51
CD50	8.31	7.27
CD51	87.12	2.49
CD54	17.49	6.22
CD61	38.45	15.59
CD73	***98.80***	***0.19***
CD90	***92.45***	***0.89***
CD105	***90.59***	***3.09***
CD106	0.47	0.43
CD112	89.39	1.90
CD144	49.02	12.66
CD146	1.82	1.11
CD166	***96.22***	***0.44***
CD271	14.87	10.84
CD304	0.85	0.75
CD325	26.81	12.96
HLA-DR	0.69	0.24

Table S2. Surface marker pattern of in vitro cultured ADMSCs.

Multicolor flow cytometry carried out surface markers' profiling of the in vitro cultured ADCMSCs. Cells showed high expression of well-known MSC markers such as CD29. CD44. CD73. CD90. and CD105 (Data shown are mean±SEM, N=14).

Top Tox Functions			
Strain	*Name*	*p-value range*	*# Molecules*
**Assays: Clinical Chemistry and Hematology**			
HSV-1 KOS	Increased Levels of Hematocrit	$5.46x10^{-06}$ - $5.46x10^{-06}$	33
	Increased Levels of Red Blood Cells	$4.79x10^{-01}$ - $1.86x10^{-04}$	33
	Decreased Levels of Albumin	$6.24x10^{-01}$ - $1.50x10^{-01}$	7
	Increased Levels of Albumin	$5.58x10^{-01}$ - $1.50x10^{-01}$	4
	Increased Levels of Alkaline Phosphatase	$2.01x10^{-01}$ - $1.53x10^{-01}$	17
HSV-1- 532	Increased Levels of Hematocrit	$5.24x10^{-03}$ - $5.24x10^{-03}$	13
	Increased Levels of Red Blood Cells	$2.13x10^{-01}$ - $3.96x10^{-02}$	12
	Decreased Levels of Albumin	$3.02x10^{-01}$ - $5.82x10^{-02}$	1
	Increased Levels of Albumin	$5.13x10^{-01}$ - $5.82x10^{-02}$	2
	Increased Levels of Alkaline Phosphatase	$2.59x10^{-01}$ - $1.33x10^{-01}$	8
HSV-2	Increased Levels of ALT	$9.26x10^{-02}$ - $9.26x10^{-02}$	1
	Increased Levels of Alkaline Phosphatase	$2.26x10^{-01}$ - $2.26x10^{-01}$	1
**Cardiotoxicity**			
HSV-1 KOS	Cardiac Enlargement	$1.00x10^{00}$ - $1.08x10^{-03}$	159
	Cardiac Infarction	$1.00x10^{00}$ - $1.29x10^{-03}$	79
	Congenital Heart Anomaly	$1.00x10^{00}$ - $3.85x10^{-03}$	83
	Cardiac Hypoplasia	$4.79x10^{-01}$ - $5.66x10^{-03}$	17
	Cardiac Necrosis/Cell Death	$5.25x10^{-01}$ - $1.21x10^{-02}$	65
HSV-1- 532	Cardiac Enlargement	$1.00x10^{00}$ - $3.38x10^{-03}$	55
	Cardiac Necrosis/Cell Death	$1.00x10^{00}$ - $9.75x10^{-03}$	24
	Cardiac Dilation	$1.00x10^{00}$ - $1.45x10^{-02}$	26
	Cardiac Inflammation	$1.00x10^{00}$ - $1.56x10^{-02}$	12
	Cardiac Proliferation	$2.82x10^{-01}$ - $1.68x10^{-02}$	17
HSV-2	Cardiac Dilation	$4.74x10^{-01}$ - $3.23x10^{-03}$	3
	Cardiac Enlargement	$4.74x10^{-01}$ - $3.23x10^{-03}$	4
	Cardiac Proliferation	$7.54x10^{-02}$ - $1.67x10^{-02}$	3
	Cardiac Dysfunction	$2.56x10^{-02}$ - $2.56x10^{-02}$	1
	Cardiac Stenosis	$1.77x10^{-01}$ - $3.18x10^{-02}$	1
**Hepatotoxicity**			
HSV-1 KOS	Liver Hyperplasia/Hyperproliferation	$1.00x10^{00}$ - $1.69x10^{-37}$	1617
	Hepatocellular carcinoma	$5.10x10^{-01}$ - $2.11x10^{-14}$	489
	Liver Necrosis/Cell Death	$4.79x10^{-01}$ - $1.19x10^{-04}$	78
	Liver Inflammation/Hepatitis	$1.00x10^{00}$ - $1.22x10^{-04}$	116
	Liver Fibrosis	$1.00x10^{00}$ - $1.76x10^{-04}$	107
HSV-1- 532	Liver Hyperplasia/Hyperproliferation	$1.00x10^{00}$ - $1.55x10^{-16}$	638
	Hepatocellular carcinoma	$1.00x10^{00}$ - $4.77x10^{-05}$	183
	Liver Inflammation/Hepatitis	$1.00x10^{00}$ - $6.83x10^{-05}$	44
	Liver Steatosis	$1.00x10^{00}$ - $6.83x10^{-05}$	50
	Liver Necrosis/Cell Death	$3.47x10^{-01}$ - $1.28x10^{-04}$	36
HSV-2	Liver Inflammation/Hepatitis	$2.18x10^{-01}$ - $1.25x10^{-08}$	11
	Liver Steatosis	$2.18x10^{-01}$ - $1.25x10^{-08}$	10
	Liver Necrosis/Cell Death	$1.13x10^{-01}$ - $1.98x10^{-03}$	5
	Liver Damage	$1.08x10^{-01}$ - $2.24x10^{-03}$	5
	Liver Failure	$3.23x10^{-03}$ - $3.23x10^{-03}$	1
Nephrotoxicity			
HSV-1 KOS	Renal Necrosis/Cell Death	$1.00x10^{00}$ - $7.51x10^{-06}$	152
	Renal Proliferation	$1.00x10^{00}$ - $2.94x10^{-05}$	80
	Glomerular Injury	$1.00x10^{00}$ - $4.22x10^{-04}$	125
	Renal Inflammation	$1.00x10^{00}$ - $4.22x10^{-04}$	87
	Renal Nephritis	$1.00x10^{00}$ - $4.22x10^{-04}$	87
HSV-1- 532	Glomerular Injury	$1.00x10^{00}$ - $1.96x10^{-04}$	58
	Renal Inflammation	$4.51x10^{-01}$ - $1.96x10^{-04}$	43

	Renal Nephritis	$4.51x10^{-01}$ - $1.96x10^{-04}$	43
	Kidney Failure	$1.00x10^{00}$ - $5.98x10^{-04}$	28
	Renal Fibrosis	$1.65x10^{-01}$ - $5.98x10^{-04}$	19
HSV-2	Glomerular Injury	$5.58x10^{-01}$ - $7.18x10^{-06}$	9
	Renal Inflammation	$3.18x10^{-02}$ - $7.18x10^{-06}$	9
	Renal Nephritis	$3.18x10^{-02}$ - $7.18x10^{-06}$	9
	Renal Damage	$6.87x10^{-02}$ - $7.54x10^{-04}$	3
	Renal Tubule Injury	$6.87x10^{-02}$ - $7.54x10^{-04}$	3

Table S3. Top TOX functions determined by IPA

**Top Regulator Effect Networks**				
Strain	*ID*	*Regulators*	*Disease & Functions*	*Consistency Score*
HSV1-KOS	1	SPDEF	Migration of cells	3.5
	2	Eldr	Cell proliferation of tumor cell lines	3.464
	3	LARP1	Cell death of tumor	3.328
	4	LARP1	Cell death of tumor cells	3.328
	5	LARP1	Necrosis of tumor	3.328
HSV1- 532	1	poly rI:rC-RNA	Replication of Herpesviridae	3.474
	2	IRF3	Replication of Herpesviridae	3.317
	3	IFNA2	Replication of Herpesviridae	3.175
	4	IRF7	Transactivation	3.162
	5	IRF7	Transactivation of RNA	3.162
HSV-2	1	SP110	Viral Infection	3.015
	2	IFNG	Cell death of epithelial cells	3.0
	3	IRF3	Cell death of epithelial cells	3.0
	4	Interferon alpha	Cell death of epithelial cells	3.0
	5	NONO	Cell death of epithelial cells	3.0

**Top Networks**			
HSV1-KOS	*ID*	*Associated Network Functions*	*Score*
	1	Cell Death and Survival. Cardiovascular Disease. Cellular Movement	35
	2	Cell Morphology. Cellular Function. and Maintenance. Metabolic Disease	35
	3	Cancer. Cell Death. and Survival. Organismal Injury. and Abnormalities	35
	4	Post-Translational Modification. Protein Folding. Developmental Disorder	35
	5	Cell-To-Cell Signaling and Interaction. Cellular Assembly and Organization. Tissue Development	33
HSV1- 532	1	Cellular Assembly and Organization. DNA Replication. Recombination. and Repair. Cellular Function and Maintenance	50
	2	Cellular Assembly and Organization. Cellular Function. and Maintenance. Molecular Transport	47
	3	RNA Damage and Repair. Cellular Function and Maintenance. Dermatological Diseases. and Conditions	47
	4	RNA Post- Transcriptional Modification. Cancer. DNA Replication. Recombination. and Repair	44
	5	RNA Post- Transcriptional Modification. Endocrine System Disorders. Hereditary Disorder	44
HSV2	1	Immunological Disease. Inflammatory Disease. Organismal Injury. and Abnormalities	45
	2	Antimicrobial Response. Inflammatory Response. Cell Signaling	36
	3	Antimicrobial Response. Inflammatory Response. Hereditary Disorder	34
	4	Immunological Disease. Inflammatory Disease. Organismal Injury. and Abnormalities	28
	5	Cell-To-Cell Signaling and Interaction. Cellular Growth and Proliferation. Connective Tissue Development. and Function	19

**Top Tox Lists**			
HSV1-KOS	Name	p-value	Overlap
	Mitochondrial Dysfunction	6.00×10^{-12}	36.3 % 62/171
	Renal Necrosis/Cell Death	2.30×10^{-08}	23.1 % 152/659
	Liver Necrosis/Cell Death	1.39×10^{-04}	22.5 % 78/347

	Recovery from Ischemic Acute Renal Failure (Rat)	3.33x10^{-04}	57.1 % 8/14
	Aryl Hydrocarbon Receptor Signaling	3.80x10^{-04}	25.5 % 41/161
HSV1- 532	Renal Necrosis/Cell Death	1.34x10^{-04}	9.4 % 62/659
	Liver Necrosis/Cell Death	5.75x10^{-04}	10.4 % 36/347
	Mitochondrial Dysfunction	9.76x10^{-04}	12.3 % 21/171
	Recovery from Ischemic Acute Renal Failure (Rat)	7.12x10^{-03}	28.6 % 4/14
	Increases Cardiac Proliferation	1.10x10^{-02}	12.8 % 11/86
HSV2	Increases Renal Nephritis	6.23x10^{-05}	6.1 % (4/66)
	Increases Liver Hepatitis	3.36x10^{-04}	3.9 % (4/102)
	Increases Depolarization of Mitochondria and Mitochondrial Membrane	1.52x10^{-03}	11.1 % (2/18)
	Liver Necrosis/Cell Death	5.33x10^{-03}	1.4 % (5/347)
	Increases Glomerular Injury	1.75x10^{-02}	1.8 % (3/169)
**Top Analysis-Ready Molecules**			
**Expr Log Ratio**			
HSV1-KOS	Molecules	Expr. Value	Chart
	CH507_528H121	18.890	↑
	CXCL11	10.522	↑
	RP3_453C1214	9.921	↑
	PRAMEF1 (includes others)	9.282	↑
	CCDC194	9.279	↑
	SOX3	9.120	↑
	ZSCAN4	8.982	↑
	CXCL10	8.889	↑
	DIO3	8.811	↑
	RP11_235E173	8.755	↑
	TMEM205	-5.128	↓
	GPR68	-4.857	↓
	SCARA3	-4.829	↓
	GREM2	-4.816	↓
	ISLR	-4.570	↓
	PSAT1	-4.459	↓
	EMILIN1	-4.235	↓
	ZER1	-4.177	↓
	SIL1	-4.170	↓
	MORN2	-4.139	↓
HSV1-532	CH507_528H121	20.970	↑
	CXCL11	10.067	↑
	CXCL10	8.526	↑
	OASL	8.087	↑
	CCDC194	7.748	↑
	UNCX	7.579	↑
	RP3_453C1214	7.339	↑
	DIO3	7.231	↑
	USP18	6.974	↑
	RSAD2	6.936	↑
	BRD3OS	-2.800	↓
	GREM2	-2.784	↓
	TMEM37	-2.295	↓
	CDK5	-2.286	↓
	RPS6KA2	-2.171	↓
	ITGB1BP1	-2.154	↓
	PTPN9	-2.059	↓
	TMEM35A	-2.047	↓
	GSTA4	-2.044	↓

	PLPP3	-2.013	↓
HSV2	CXCL11	9.671	↑
	CH507_528H121	9.041	↑
	CXCL10	7.910	↑
	OASL	7.060	↑
	IDO1	6.643	↑
	MX1	6.268	↑
	CMPK2	6.250	↑
	CCDC194	6.106	↑
	IFIT2	5.878	↑
	RSAD2	5.534	↑

Table S4. Top networks and analysis-ready molecules determined by IPA. Green arrows indicate downregulation and red arrows indicate upregulation of genes.

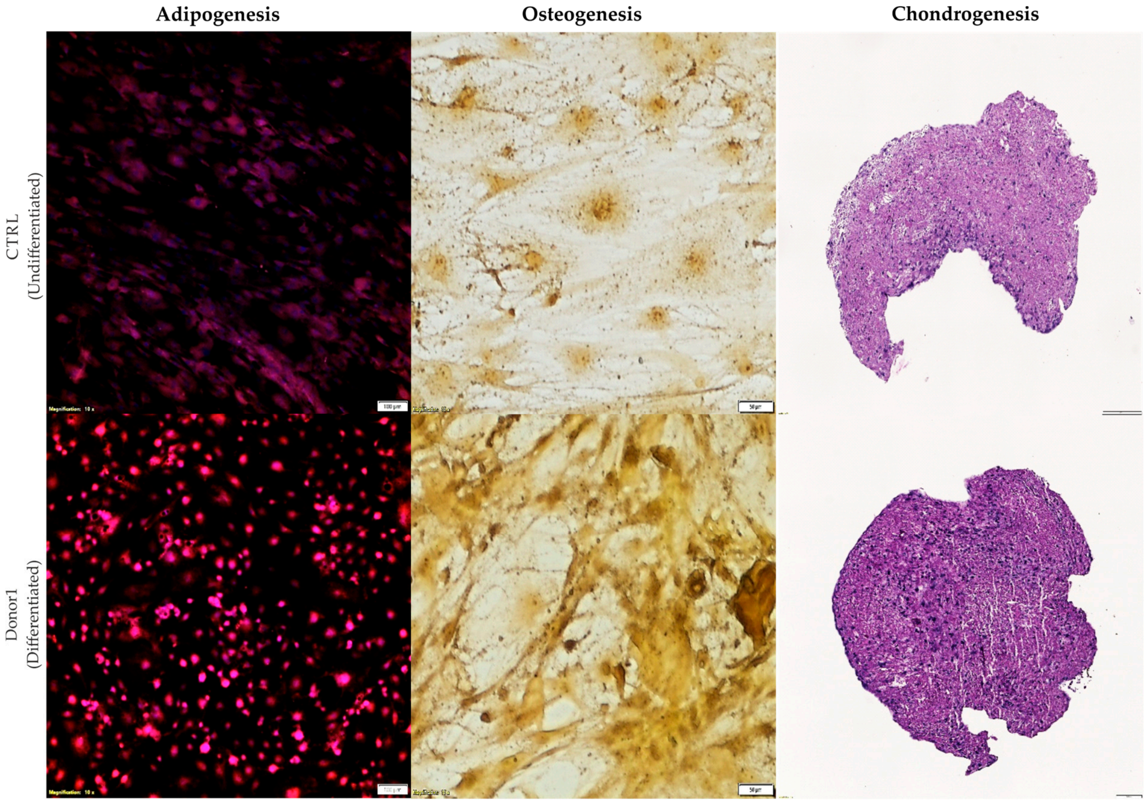

Figure S1. Validation of the in vitro differentiation potential of AD-MSC
MSCs isolated from adipose tissue were able to differentiate into adipose tissue in vitro. as revealed by Nile-red staining of lipid droplets in the cells. In the case of bone-directed differentiation. calcium deposits were detected by alizarin red staining. The extracellular matrix appearance characteristic of cartilage tissue was shown by metachromasia of toluidine blue staining. Results from a representative study are shown.(CT: control culture without differentiation cell culture media). Scale bar: For Adipogenesis 100um, for Osteogenesis 50 um and for chondrogenesis 200um.

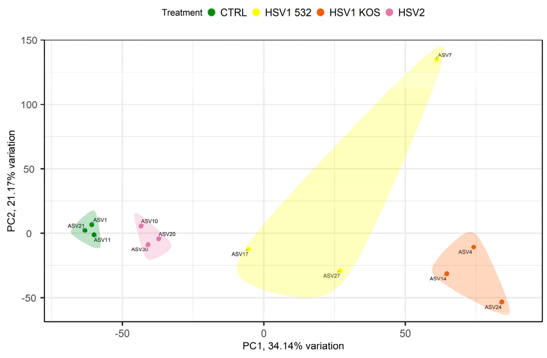
Treatment
CTRL
HSV1 532
HSV1 KOS
HSV2
ASV1
ASV21
ASV11
ASV10
ASV20
ASV30
ASV17
ASV27
ASV7
ASV4
ASV14
ASV24
150
100
50
0
-50
PC2, 21.17% variation
PC1, 34.14% variation

A

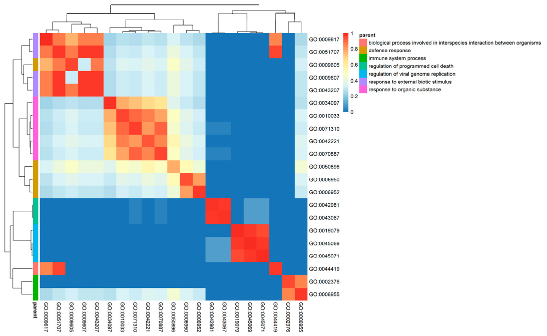
GO:0009617
GO:0051707
GO:0009605
GO:0009607
GO:0043207
GO:0034097
GO:0010033
GO:0071310
GO:0042221
GO:0070887
GO:0050896
GO:0006950
GO:0006952
parent
biological process involved in interspecies interaction between organisms
defense response
immune system process
regulation of programmed cell death
regulation of viral genome replication
response to external biotic stimulus
response to organic substance

B

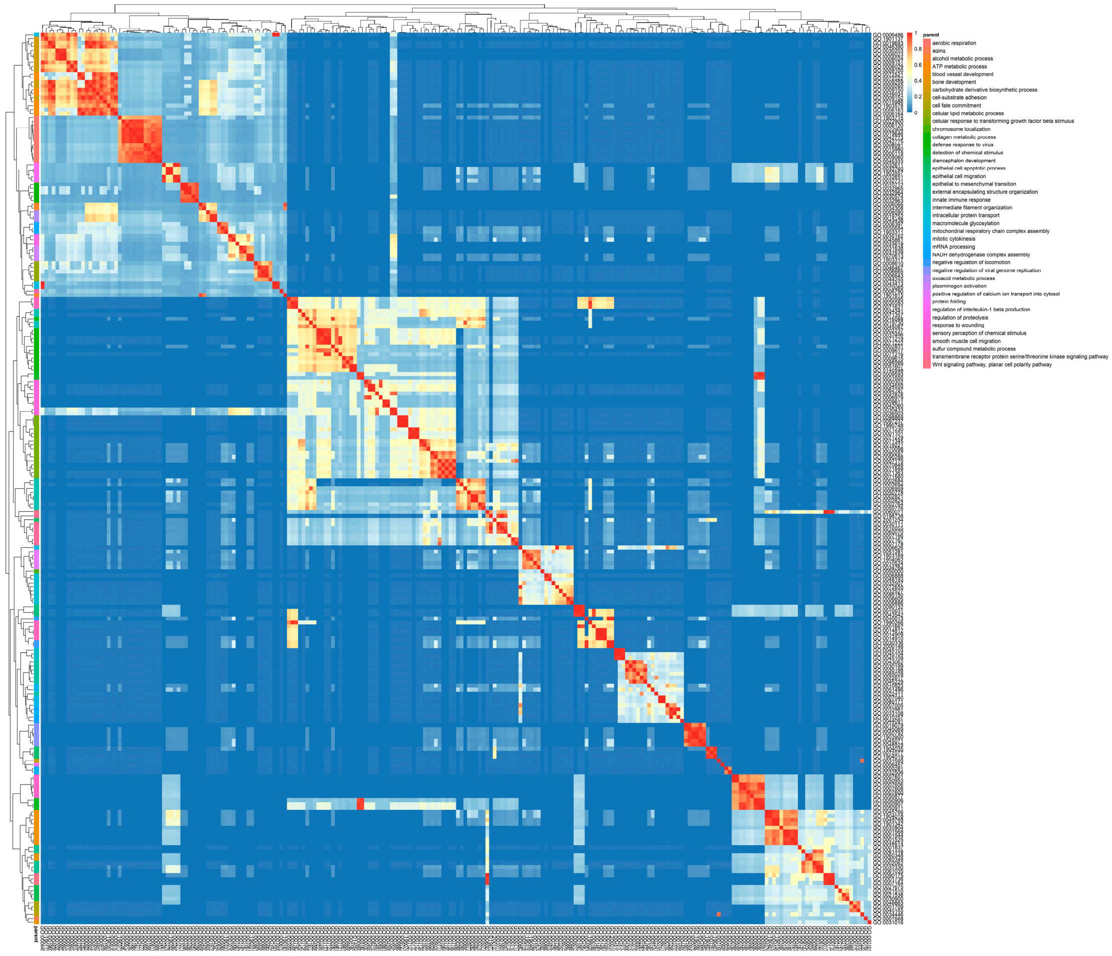
parent
aerobic respiration
aging
alcohol metabolic process
ATP metabolic process
blood vessel development
bone development
carbohydrate derivative biosynthetic process
cell-substrate adhesion
cell fate commitment
cellular lipid metabolic process
cellular response to transforming growth factor beta stimulus
chromosome localization
collagen metabolic process
defense response to virus
detection of chemical stimulus
diencephalon development
epithelial cell apoptotic process
epithelial cell migration
epithelial to mesenchymal transition
external encapsulating structure organization
innate immune response
intermediate filament organization
intracellular protein transport
macromolecule glycosylation
mitochondrial respiratory chain complex assembly
mitotic cytokinesis
mRNA processing
NADH dehydrogenase complex assembly
negative regulation of locomotion
negative regulation of viral genome replication
oxoacid metabolic process
plasminogen activation
positive regulation of calcium ion transport into cytosol
protein folding
regulation of interleukin-1 beta production
regulation of proteolysis
response to wounding
sensory perception of chemical stimulus
smooth muscle cell migration
sulfur compound metabolic process
transmembrane receptor protein serine/threonine kinase signaling pathway
Wnt signaling pathway, planar cell polarity pathway

C

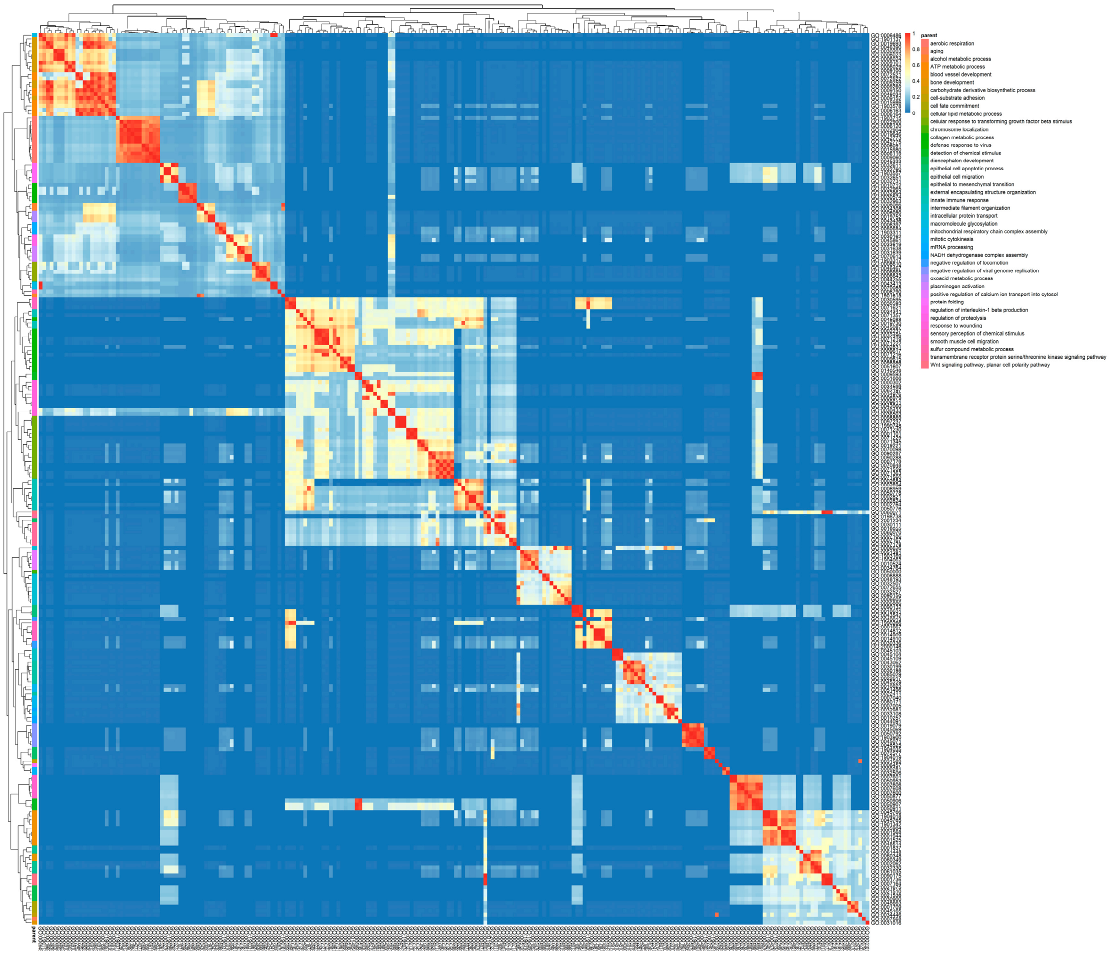

D

Figure S2. QC of RNASeq samples used in the study.

PC Plot of samples showed different clusters according to the treatments. (A) GSEA analysis of all data sets. The figures show a heat map of GSEA scores for the GO categories selected in the rank-based analysis (B-D).

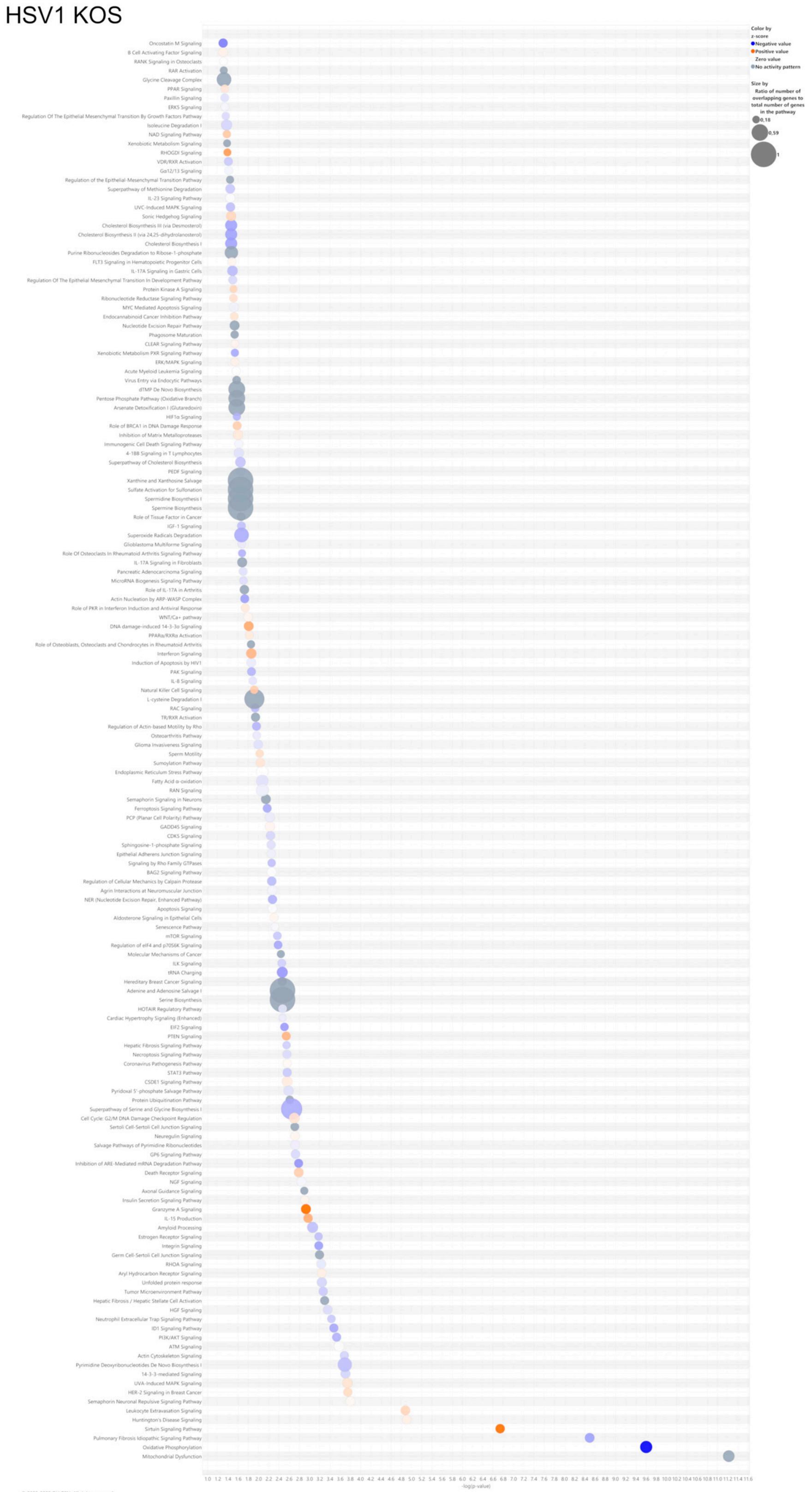

© 2000-2023 QIAGEN. All rights reserved.

Figure S3. Canonical pathways in HSV-1 KOS infected ADMSC

Bubble chart of canonical pathways initiated by HSV-1 KOS infection in vitro. Pathways are determined by IPA.

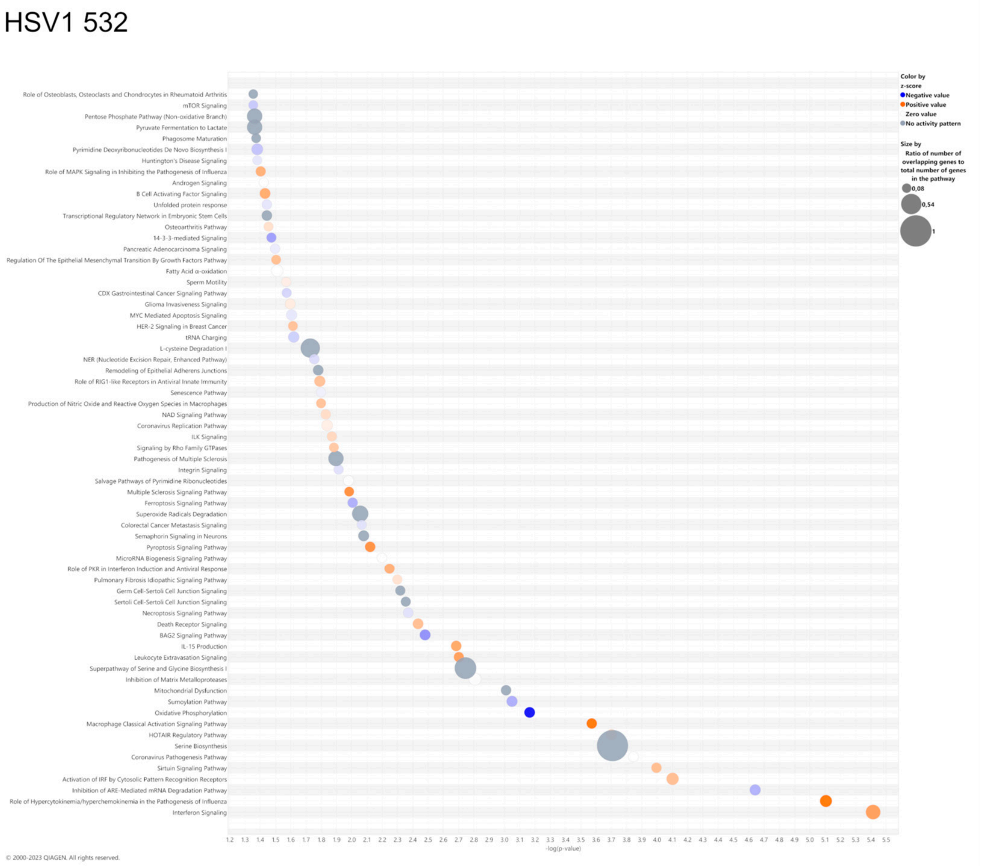

Figure S4. Canonical pathways in HSV-1 532 infected ADMSC

Bubble chart of canonical pathways initiated by HSV-1 532 infection in vitro. Pathways are determined by IPA.

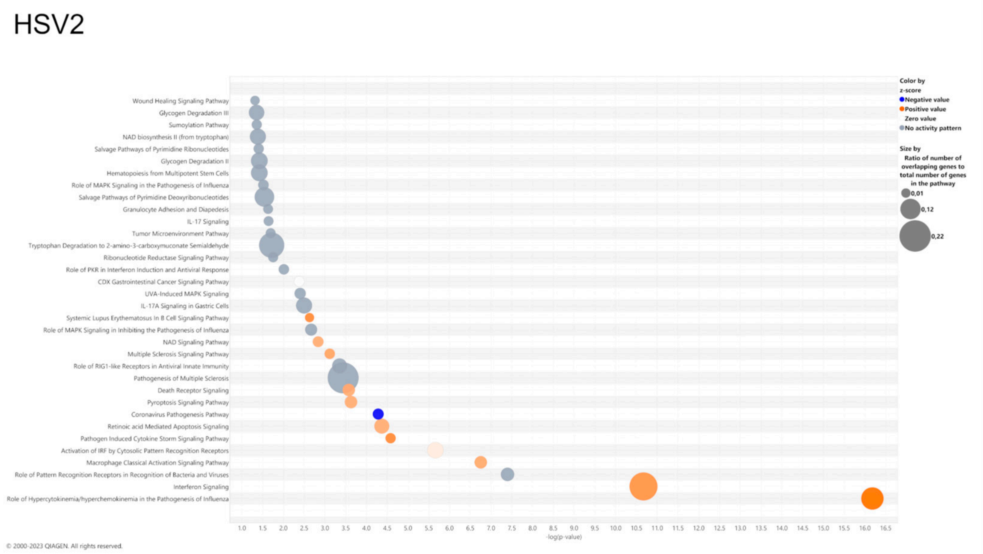

Figure S5. Canonical pathways in HSV-2 infected ADMSC

Bubble chart of canonical pathways initiated by HSV-2 infection in vitro. Pathways are determined by IPA.

HSV1 KOS

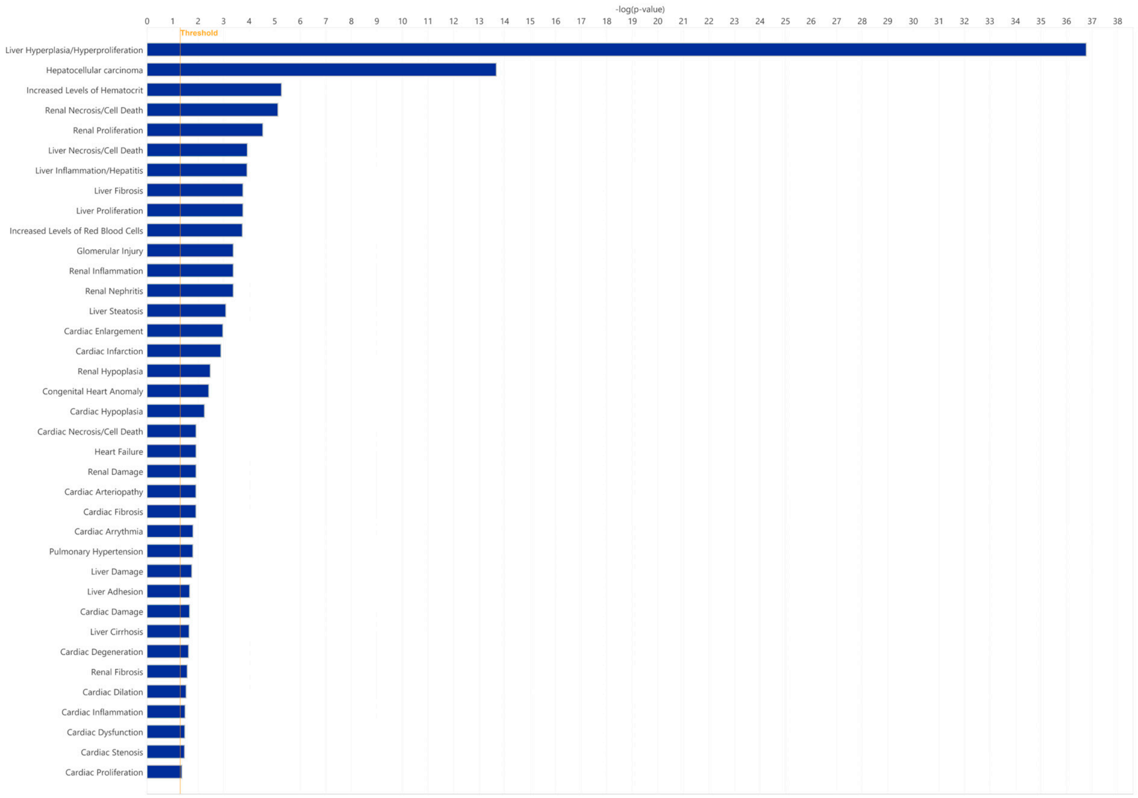

Figure S6. TOX pathways in HSV-1 KOS infected ADMSC

Bar chart of TOX pathways activated by in vitro HSV-1 KOS infection in AD-MSC. Pathways were determined by IPA.

HSV1 532

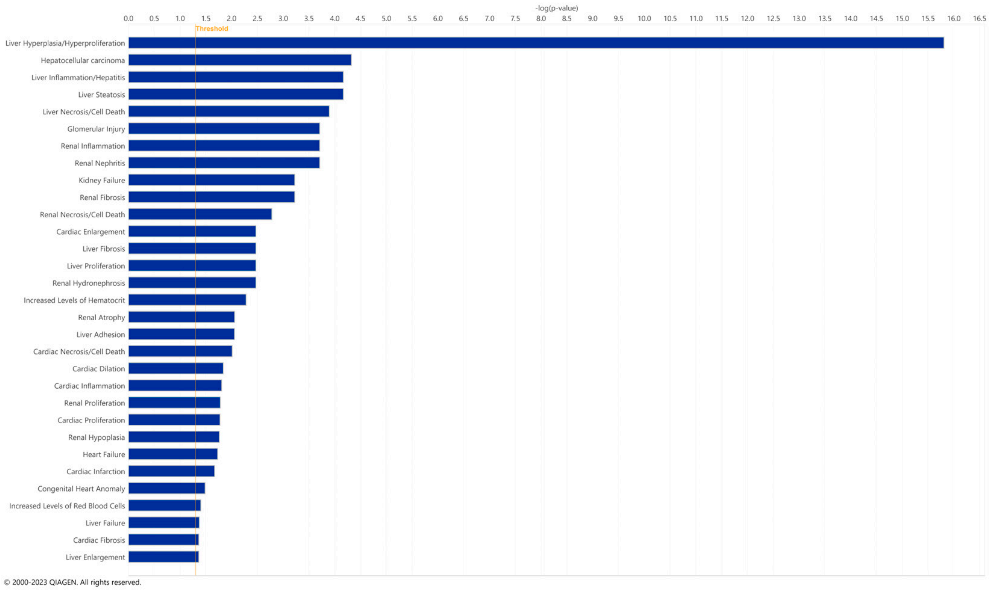

HSV2

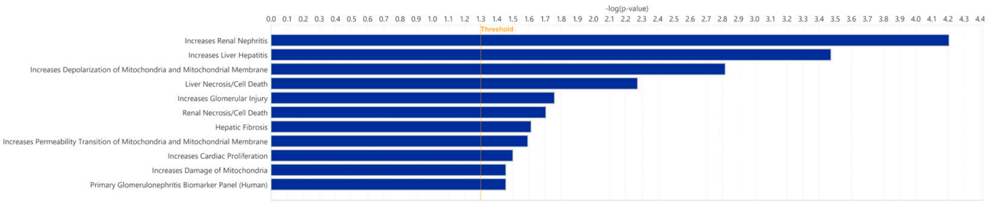

Figure S7. TOX pathways in HSV-1 532 and HSV-2 infected ADMSC
Bar chart of TOX pathways activated by in vitro HSV-1 532 or HSV-2 infection in AD-MSC. Pathways were determined by IPA.

A

	1.-2.	3.-4.	5.-6.	7.-8.	9.-10.	11.-12.
A	(+) control	Adiponectin	Aggrecan	Angiogenin	Angiopoietin-1	Angiopoietin-2
B		CD40 ligand	Chitinase 3-like 1	Complement Factor D	C-Reactive Protein	Cripto-1
C		ENA-78	Endoglin	Fas Ligand	FGF basic	FGF-7
D	GRO-α	Growth Hormone	HGF	ICAM-1	IFN-γ	IGFBP-2
E	IL-4	IL-5	IL-6	IL-8	IL-10	IL-11
F	IL-19	IL-22	IL-23	IL-24	IL-27	IL-31
G	Leptin	LIF	Lipocalin-2	MCP-1	MCP-3	M-CSF
H	Myeloperoxidase	Osteopontin	PDGF-AA	PDGF-AB/BB	Pentraxin-3	PF4
I	Serpin E1	SHBG	ST2	TARC	TFF3	TfR
J	(+) control		Vitamin D BP			
	13.-14.	15.-16.	17.-18.	19.-20.	21.-22.	23.-24.
A	BAFF	BDNF	Complement Component C5/C5a	CD14	CD30	(+) control
B	Cystatin C	Dkk-1	DPPIV	EGF	EMMPRIN	
C	FGF-19	Flt-3 Ligand	G-CSF	GDF-15	GM-CSF	
D	IGFBP-3	IL-1α	IL-1β	IL-1ra	IL-2	IL-3
E	IL-12, p70	IL-13	IL-15	IL-16	IL-17A	IL-18 Bpa
F	IL-32α/β/γ	IL-33	IL-34	IP-10	I-TAC	Kallikrein 3
G	MIF	MIG	MIP-1α/MIP-1β	MIP-3α	MIP-3β	MMP-9
H	RAGE	RANTES	RBP4	Relaxin-2	Resistin	SDF-1α
I	TGF-α	Thrombospondin-1	TNF-alpha	uPAR	VEGF	
J						(-) control

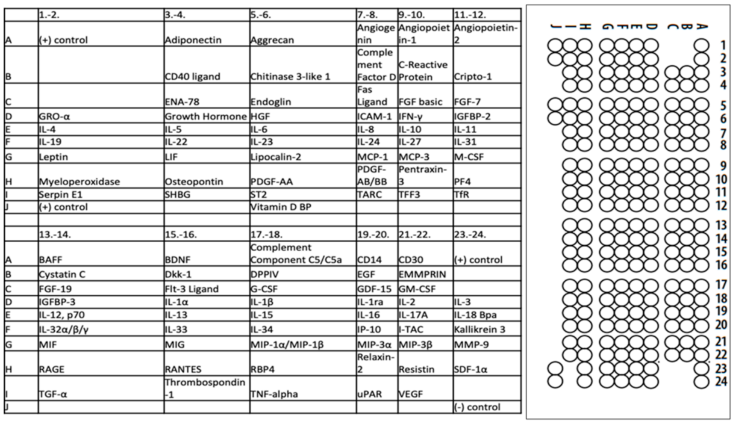

B

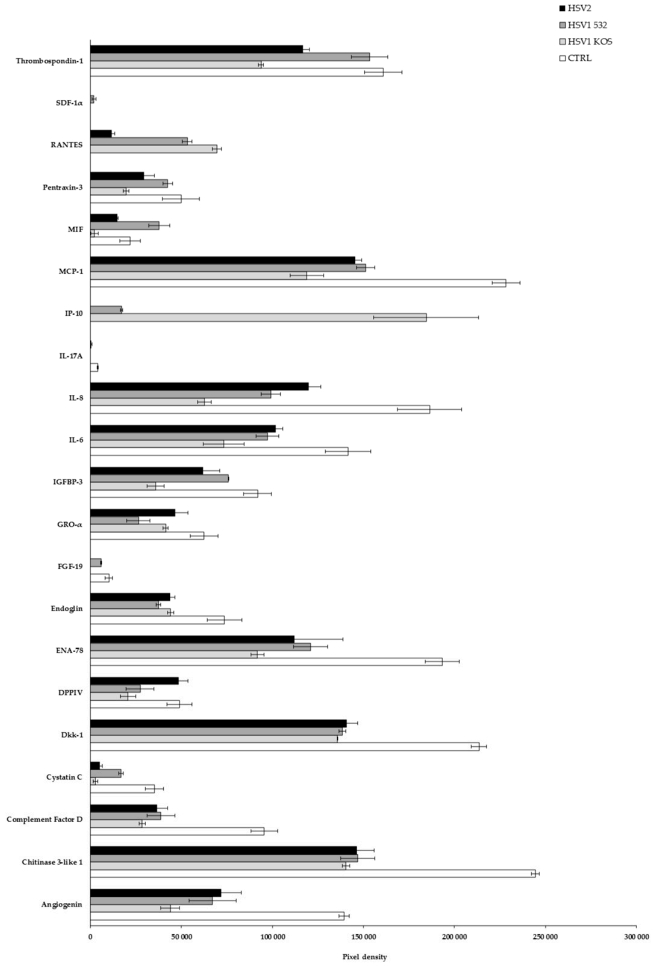

C

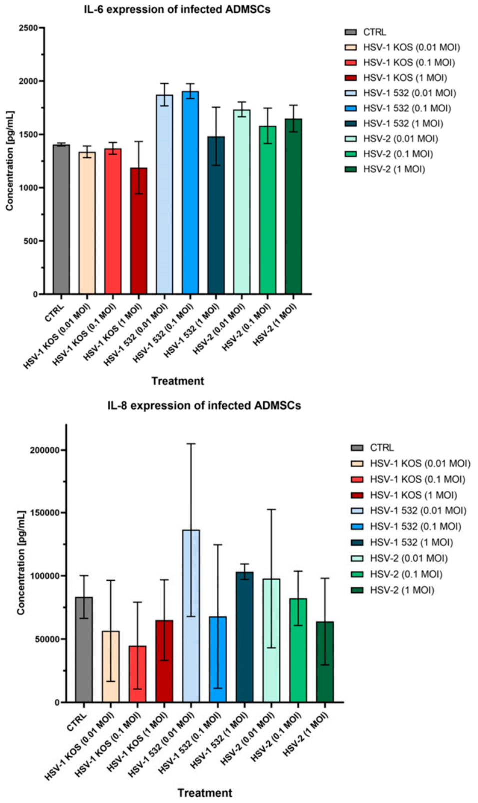

Figure S8. Expression of cytokines released by HSV-infected and uninfected AD-MSC

The setup of the Human XL Cytokine Proteome Profiler to identify targets in the cell culture supernatants (**A**). All spots were analyzed and semi-quantified by image analysis. (**B**) IL-6, IL-8 and IP-10 secretion were validated by ELISA. (N=3) **(C)**
